# Incidence of type 2 diabetes and metabolic syndrome by Occupation – 10-Year follow-up of the Gutenberg Health Study

**DOI:** 10.1186/s12889-025-21732-5

**Published:** 2025-02-07

**Authors:** Juliane Bauer, Janice Hegewald, Karin Rossnagel, Sylvia Jankowiak, Michaela Prigge, Julian Chalabi, Matthias Nübling, Alice Freiberg, Merle Riechmann-Wolf, Pavel Dietz, Philipp S. Wild, Thomas Koeck, Manfred E. Beutel, Norbert Pfeiffer, Karl J. Lackner, Thomas Münzel, Konstantin Strauch, Philipp Lurz, Oliver Tüscher, Julia Weinmann-Menke, Stavros Konstantinides, Andreas Seidler

**Affiliations:** 1 Division Work and Health, Federal Institute for Occupational Safety and Health (BAuA), Berlin, Germany; 2 Preventive Cardiology and Preventive Medicine, Department of Cardiology, University Medical Center of the Johannes Gutenberg University Mainz, Mainz, Germany; 3 FFAW: Freiburg Research Centre for Occupational Sciences, Freiburg, Germany; 4 Institute and Policlinic of Occupational and Social Medicine (IPAS), Faculty of Medicine, TU Dresden, Dresden, Germany; 5 Institute of Occupational, Social, and Environmental Medicine, University Medical Center of the Johannes Gutenberg University Mainz, Mainz, Germany; 6 Center for Thrombosis and Hemostasis (CTH), University Medical Center of the Johannes Gutenberg University Mainz, Mainz, Germany; 7 German Center for Cardiovascular Research (DZHK), Partner Site Rhine-Main, Mainz, Germany; 8 Institute of Molecular Biology (IMB), Mainz, Germany; 9 Department of Psychosomatic Medicine and Psychotherapy, University Medical Center of the Johannes Gutenberg University Mainz, Mainz, Germany; 10 Department of Ophthalmology, University Medical Center of the Johannes Gutenberg University Mainz, Mainz, Germany; 11 Institute for Clinical Chemistry and Laboratory Medicine, University Medical Center of the Johannes Gutenberg University Mainz, Mainz, Germany; 12 Department of Cardiology – Cardiology I, University Medical Center of the Johannes Gutenberg University Mainz, Mainz, Germany; 13 Institute of Medical Biostatistics, Epidemiology and Informatics (IMBEI), University Medical Center of the Johannes Gutenberg University Mainz, Mainz, Germany; 14 Department of Psychiatry and Psychotherapy, University Medical Center of the Johannes Gutenberg University Mainz, Mainz, Germany; 15 Department of Nephrology and Rheumatology, Center of Immunotherapy, University Medical Center of the Johannes Gutenberg-University Mainz, Mainz, Germany

**Keywords:** Cardiometabolic, Epidemiology, Incidence, Longitudinal, Metabolic syndrome, Occupation, Population-based, Prevalence, Prevention, Type 2 diabetes

## Abstract

**Background:**

In view of demographic change, rising retirement age, and a growing shortage of skilled workers, it is increasingly important to prevent widespread diseases such as type 2 diabetes or its risk factor metabolic syndrome. Since the workplace is an important setting for preventive measures and little is known about incident cases in the working population, the aim of this study was to identify vulnerable occupational groups for whom these interventions are particularly appropriate. Therefore, we investigated the 10-year incidence of type 2 diabetes and metabolic syndrome across occupational groups in Germany.

**Methods:**

Employees of the population-based Gutenberg-Health-Study (GHS) were examined at baseline (2007–2012) and 10 years later. We calculated age- and sex-standardised incidence rates and standardised incidence ratios (SIR) with a 95% confidence interval (CI) for occupations, job complexity levels, and supervisory and managerial positions. 5954 persons at risk for type 2 diabetes and 5103 at risk for metabolic syndrome were observed.

**Results:**

Between baseline and follow-up, 388 cases of type 2 diabetes and 1104 cases of metabolic syndrome occurred, and standardised incidences were 6.9% and 22.6%, respectively. The highest incidence of type 2 diabetes was observed in the occupational group “food production and processing” (20.7%) with a threefold increased incidence (SIR = 3.0, 95% CI 1.8–4.7) compared to the total working population of the GHS. Employees in “metal production, processing and construction” had the highest incidence of metabolic syndrome and a two times higher SIR (48.5%; SIR = 2.1, 95% CI 1.4–2.9). There was also a high incidence of both type 2 diabetes and metabolic syndrome in “cleaners” (16.5% and 34.8%) and “drivers and mobile plant operators” (14.8% and 41.2%). An increased incidence of type 2 diabetes and metabolic syndrome was observed with decreasing job complexity levels.

**Conclusions:**

This study shows wide differences in the incidence of type 2 diabetes and metabolic syndrome between occupational groups and highlights the vulnerability of certain occupations. As the workplace is an important platform for interventions, the findings of this study could guide the development of more nuanced and effective workplace health initiatives to promote a healthier workforce for the future.

**Supplementary Information:**

The online version contains supplementary material available at 10.1186/s12889-025-21732-5.

## Introduction

Type 2 diabetes and its major risk factor, metabolic syndrome, remain a global burden on healthcare systems due to treatment costs [1], high mortality rates [2] as well as incapacity for work, and early retirement [3]. According to the International Diabetes Federation, globally 10.5% of the adult population (20–79 years) were affected in 2021 [2]. In Germany, the proportion of diabetes cases (type 1 and 2) in adults aged 18 to 79 is estimated to be around 7.2% [4]. By 2040, the prevalence of type 2 diabetes among adults in Germany is predicted to increase by 54 to 77% [5]. In addition, in the year 2019, one in four people (26%) in Germany met the criteria for metabolic syndrome (abdominal obesity, hypertension, lipid metabolism disorder and impaired glucose tolerance) [6]. The average age of diabetes diagnosis (type 1 and 2) is currently around 53 years, an age at which most people are typically still working, highlighting the need to consider type 2 diabetes and metabolic syndrome in an occupational context [7]. In addition to lifestyle-related risk factors, the workplace can be both a risk factor and an opportunity for preventing type 2 diabetes and metabolic syndrome. Therefore, the workplace represents an important setting for health promotion and screenings that can identify undiagnosed diseases such as diabetes, obesity, and hypertension [8, 9]. To implement or strengthen targeted prevention measures, it is important to identify occupations with an increased risk. A systematic review showed that metabolic syndrome is a common health risk factor in all occupational groups and that the prevalence varies widely between countries and occupational groups, ranging from 5.2 to 39.2% [10]. Previous studies have examined the association between type 2 diabetes or metabolic syndrome and occupation cross-sectionally [11–15]. However, the extent to which new cases occur in different occupations cannot be estimated from cross-sectional studies. In addition, an examination of incidence can identify vulnerable occupational groups that need more prevention. To date, only one study is known that investigated the incidence of type 2 diabetes or metabolic syndrome in different occupational groups [16, 17]. Recently, one study identified differences in type 2 diabetes prevalence among the occupational areas in Germany using health insurance records [18]. However, the different occupational groups within broader occupational areas are varied, so occupations should be considered as differentiated as possible in order to identify occupations for preventive measures.

To address this research gap and enhance the current body of evidence regarding longitudinal studies, this study aims to examine the 10-year incidence of type 2 diabetes and metabolic syndrome among employees in Germany. Data from the Gutenberg-Health-Study (GHS) were used to identify occupational groups with an increased incidence.

## Methods

### Design and participants

We used longitudinal data from the GHS. The GHS is a population-based, prospective, observational single-centre cohort study in the Rhine-Main-Region in Germany.

The original focus of the GHS was to analyse cardiovascular risk factors and improve risk prediction for corresponding diseases [19]. Written informed consent was obtained from all participants. The local ethics committee and the local and federal data safety commissioners approved the study (#837.020.07(5555)). Participants were selected randomly from the local registry of the city of Mainz and the district of Mainz-Bingen. The random sample was stratified 1:1 for sex, residence (urban and rural areas), and age. Inclusion criteria were written informed consent and age between 35 and 74 years. Persons with insufficient German language knowledge were not included in the study, as well as persons who could not come to the study centre due to physical or mental impairment. A detailed description of the design and the rationale of the GHS is published elsewhere [19].

A total of 15,010 participants (response rate 60.4%) were enrolled in the baseline examination between 2007 and 2012. At the follow-up 10 years later (2017–2022), 9575 individuals participated (retention rate 63.8%), 963 persons died, 2303 persons declined to continue participating, 123 persons were excluded, 399 could not be contacted, and 1647 people only took part in the telephone interview.

**Fig. 1:**
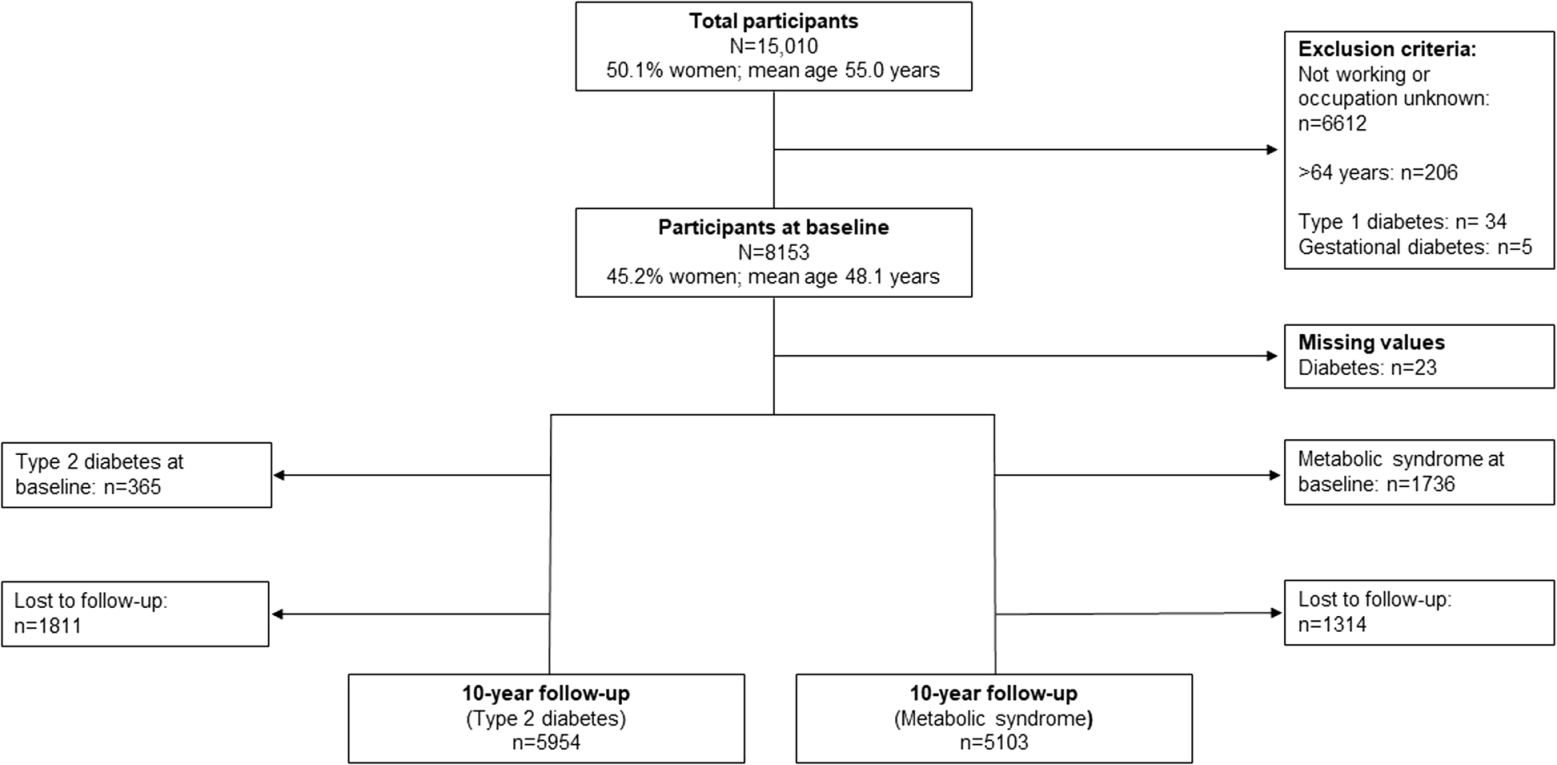
Flow chart of study population

For this study, individuals were excluded from analyses if they were (1) not employed or working at baseline or if the occupation was unknown (*n* = 6612), (2) older than 64 years of age (retirement age) at the day of the interview (*n* = 206), and (3) diagnosed with type 1 diabetes or gestational diabetes (*n* = 39). After excluding 23 participants due to missing information on diabetes, 8130 individuals were included for type 2 diabetes and 8153 for metabolic syndrome at baseline. For the examination of incidence, participants with prevalent type 2 diabetes (*n* = 365) or metabolic syndrome (*n* = 1736) at baseline were excluded, respectively. Due to lost to follow-up, 5954 persons at risk for type 2 diabetes and 5103 at risk for metabolic syndrome were ultimately observed (Fig. 1).

### Exposure and demographic variables 

Information on age, sex, educational and professional qualifications, occupational phases and the occupational variables total working time, overtime per week, and full-time employment were obtained from a computer-assisted interview (see additional file 1).

Information about previous occupational phases, including the economic sector and job description, were then coded into occupational categories according to the German Classification of Occupations (KldB, *Klassifikation der Berufe*) 2010 [20]. The KldB 2010 reflects the current professional landscape in Germany and offers high compatibility with the International Standard Classification of Occupations (ISCO) [21]. The KldB is coded in five digits and is hierarchically structured. The first digit of the code describes the occupational area, the second the main occupational group, the third the occupational group, the fourth the occupational subgroup as well as supervisory and managerial positions, and the fifth the job complexity. The job complexity reflects the requirement level in four levels: “low” (helpers and semi-skilled), “medium” (skilled workers), “high” (specialists) and “very high” (experts).

For this study, the occupation at the time of the baseline examination was used. Occupational groups were investigated at the one- and two-digit levels. The one-digit level contains 10 occupational areas, and the subsequent level is divided into 37 main occupational groups. The occupational area “military” was not considered, because the classification in this group primarily reflects the rank and not the activity. Main occupational groups with fewer than 20 individuals or less than five incident cases between baseline and 10-year follow-up are not presented.

We examined job complexity as a proxy variable for socioeconomic status (SES). Job complexity is a good indicator of SES, as complex jobs tend to require higher levels of education and provide more financial compensation.

For quality assurance, 10% of the subjects were coded twice. Interrater reliability between primary and dual coding was calculated for different dimensions of the occupational code. Overall, the interrater reliability for the various dimensions was rated as good to very good (Cohen’s kappa coefficient = 0.88, 95% CI 0.87–0.89) [22].

### Outcomes

Type 2 diabetes was defined as either a measured HbA1c-level ≥ 6.5%, answering yes to the questions “Do you have diabetes?” and “Has this been diagnosed or confirmed by a doctor?” or intake of insulin or oral glucose-lowering drugs [anatomical therapeutic chemical (ATC) code A10].

For the definition of metabolic syndrome, we followed the harmonized recommendations of Alberti et al. [23]. Participants had to fulfil at least three of the following five criteria:

1. obesity: waist circumference ≥ 80 cm for female and ≥ 94 cm for male participants.
2. triglycerides ≥ 150 mg/dl or on drug treatment for increased triglycerides (ATC code C10);
3. high-density lipoprotein cholesterol (HDL) < 50 mg/dl in women and < 40 mg/dl in men or on drug treatment for reduced HDL cholesterol (ATC code C10);
4. arterial blood pressure increased to ≥ 130 mmHg systolic or ≥ 85 mmHg diastolic or on antihypertensive drug treatment (ATC codes C02, C03, C07, C08 or C09);
5. fasting blood glucose ≥ 100 mg/dl or on drug treatment for increased glucose (ATC code A10).

Waist circumference was measured with a non-stretching tape measure midway between the lowest rib and the superior anterior iliac spine in position of expiration. Systolic and diastolic blood pressure were determined as the average of the 2nd and 3rd standardised measurement after 8 and 11 min of rest. Blood samples were drawn after an overnight fasting period of at least 8 h. Glucose, HDL cholesterol, and triglycerides were determined by routine laboratory methods. In general, all examinations were done according to standard operating procedures (SOPs) by certified medical technical assistants.

### Statistical analyses

Descriptive data of the study population are presented as numbers and percentage or mean and standard deviation. Type 2 diabetes and metabolic syndrome status were examined separately, and descriptive analyses were stratified by sex.

Age- and sex-standardised prevalence and incidence rates with 95% confidence interval (CI) were calculated for each occupational group, job complexity level, and managers and supervisors. For age- and sex-standardisation inverse probability treatment weighting [24] was used based on the age- and sex-distribution of the German population in 2021 (Federal Statistical Office of Germany) (see additional file 2). The incidence rates are expressed as percentages. Standardised incidence ratios (SIR) and 95% CI across occupations were calculated compared to the overall age- and sex-standardised incidence in the total GHS working population (reference incidence). Since the focus of this study is on the incidence of type 2 diabetes and metabolic syndrome, the prevalence across occupational groups is presented in the results but not discussed in detail.

Incidence rates were also calculated for job complexity level, and for supervisors and managers. The results were calculated for the total working population and separately for women and men. Due to the uneven representation of men and women in some occupations, several occupations could not be studied for both sexes. All analyses were conducted with R version 4.2.1 [25].

## Results

### Characteristics of the study population

Of the 8153 participants, 45.2% were female and the mean age was 48.4 years. Around half of the participants (48.1%) had the highest educational level (A-levels/high school diploma), and, regarding professional qualifications, 41.3% had a primary vocational school qualification, and 36.6% had a university degree. In addition, almost half worked in jobs with a medium job complexity level (44.6%), the other half in a job with a high or very high complexity level (21.0% and 30.8%, respectively), and only a small proportion worked in low-complexity jobs (3.6%). On average, employees had worked at their current jobs for around 14 years. Detailed information on the characteristics of the study population can be found in Table 1.

**Table 1:** Population characteristics at baseline, stratified by sex

	Total ^a^ (*n* = 8153)	Women (*n* = 3685)	Men (*n* = 4468)
**Age [years]**,** mean (SD)**	48.4 (7.6)	48.1 (7.4)	48.6 (7.8)
**Education**,** % (n)**			
Secondary general school (graduation after 9 years)	27.4 (2234)	24.8 (913)	29.6 (1321)
Intermediate school (graduation after 10 years)	23.5 (1917)	30.7 (1130)	17.6 (787)
High school diploma	48.1 (3914)	43.4 (1599)	51.9 (2315)
Other educational qualification	0.5 (41)	0.7 (24)	0.4 (17)
No qualification	0.5 (38)	0.4 (15)	0.5 (23)
**Professional qualification**,** % (n)**			
Primary vocational school	41.3 (3355)	48.6 (1790)	35.1 (1565)
Secondary vocational school	15.6 (1266)	13.4 (492)	17.4 (774)
University degree	36.6 (2976)	30.8 (1133)	41.4 (1843)
Other professional qualification	2.3 (186)	2.3 (86)	2.2 (100)
No qualification	4.3 (350)	4.9 (179)	3.8 (171)
**Total working time [hours/week]**,** mean (SD)**^**b**^	40.8 (13.0)	34.5 (12.7)	46.2 (10.7)
**Years at current work place**,** mean (SD)**	14.3 (10.5)	13.3 (10.2)	15.1 (10.6)
**Full-time work**,** % (n)**	73.3 (5830)	48.8 (1771)	93.7 (4059)
**Job complexity**,** % (n)**			
Low	3.6 (294)	5.7 (209)	1.9 (85)
Medium	44.6 (3640)	53.8 (1982)	37.1 (1658)
High	21.0 (1709)	17.6 (649)	23.7 (1060)
Very high	30.8 (2510)	22.9 (845)	37.3 (1665)
**Supervisors and managers**,** % (n)**	12.0 (976)	6.8 (3685)	16.3 (4468)
**Type 2 diabetes**,** % (n)**	4.5 (365)	3.1 (114)	5.6 (251)
**Metabolic syndrome (at least 3 of 5 criteria)**,** % (n)**	21.3 (1736)	13.2 (488)	27.9 (1248)
Central obesity	62.3 (5078)	66.1 (2434)	59.2 (2644)
High triglycerides	20.5 (1673)	12.7 (467)	27.0 (1206)
Low HDL	20.1 (1640)	11.9 (438)	26.9 (1202)
Hypertension	39.6 (3231)	33.0 (1214)	45.2 (2017)
High fasting blood glucose	11.5 (934)	5.9 (216)	14.1 (632)

^a^missing values: type 2 diabetes = 23; SES and education = 9; professional qualification = 20; total working time = 11

^b^fixed working time + overtime

### Incidence of type 2 diabetes

**Fig. 2:**
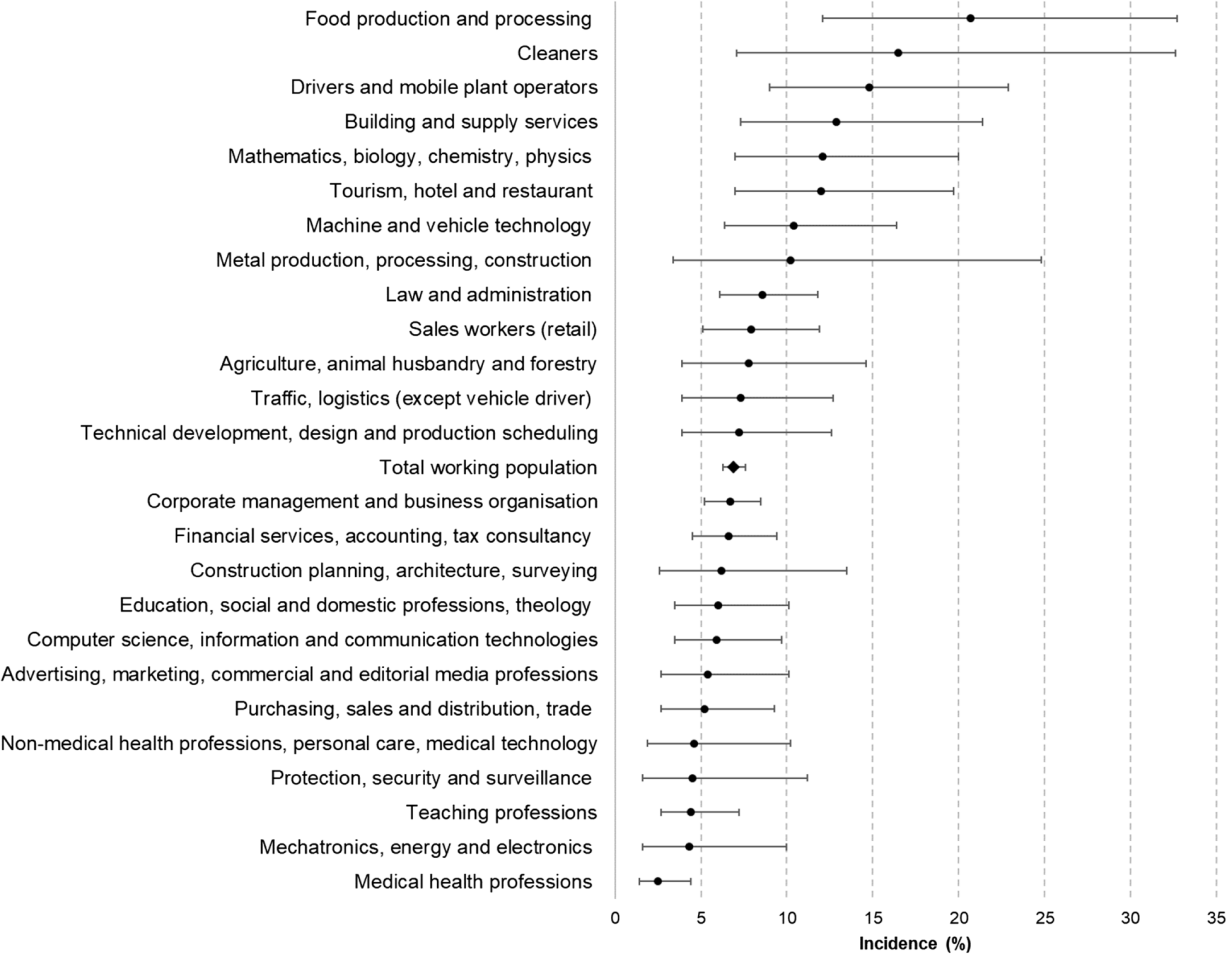
Age- and sex-standardized incidence and 95% CI of type 2 diabetes, stratified by main occupational group

Between baseline and the 10-year follow-up, a total of 388 cases occurred. Overall, age- and sex-standardised incidence of type 2 diabetes was 6.9%. Of the nine occupational areas, “construction, architecture, surveying and building technology” and “transport, logistics, protection and safety” showed the highest incidence (9.7% and 9.5%, respectively). Among the main occupational groups, the highest standardised 10-year incidence was observed among “food production and processing” workers (20.7%), “cleaners” (16.5%), and “drivers and mobile plant operators” (14.8%). The lowest incidence of type 2 diabetes was in the occupational group “medical health professions” (2.5%) (Fig. 2; Table 2). Compared to the total working population, the SIR showed a threefold increased incidence for employees in “food production and processing” (SIR = 3.0, 95% CI 1.8–4.7) and a two times higher incidence for “cleaners” (SIR = 2.2, 95% CI 1.0–4.7) and “drivers and mobile plant operators” (SIR = 2.2, 95% CI 1.3–3.3) (Table 2).

**Table 2:** Age- and sex-standardised incidence and SIR of type 2 diabetes for occupational areas and main occupational groups, job complexity level and managerial position

	*N* Baseline	Prevalent cases	Age- and sex-standardized Prevalence (95% CI)	Persons at risk	Incident cases	Age- and sex-standardized Incidence (95% CI)	SIR (95% CI)
**Total working population**	**8130**	**365**	**5.3 (4.8–5.8)**	**5954**	**388**	**6.9 (6.3–7.6)**	**–**
**Agriculture, forestry, animal husbandry and horticulture**	**229**	**7**	**3.7 (1.8–7.2)**	**170**	**10**	**6.0 (3.1–11.0)**	**0.9 (0.5–1.6)**
Agriculture, animal husbandry and forestry	151	6	4.9 (2.3–9.7)	114	9	7.8 (3.9–14.6)	1.1 (0.6–2.1)
**Raw material extraction**,** production and manufacturing**	**1095**	**70**	**7.2 (5.7–9.0)**	**775**	**62**	**8.8 (6.8–11.2)**	**1.3 (1.0–1.6)**
Metal production, processing and construction	76	6	7.9 (3.1–17.7)	47	5	10.2 (3.4–24.8)	1.5 (0.5–3.6)
Machine and vehicle technology	269	20	8.5 (5.4–13.0)	191	21	10.4 (6.4–16.4)	1.5 (0.9–2.4)
Mechatronics, energy and electronics	188	13	7.5 (4.2–12.8)	137	7	4.3 (1.6–10.0)	0.6 (0.2–1.5)
Technical development, design and production scheduling	253	14	6.2 (3.5–10.3)	189	13	7.2 (3.9–12.6)	1.1 (0.6–1.8)
Food production and processing	98	7	8.2 (4.0–15.7)	63	9	20.7 (12.1–32.7)	3.0 (1.8–4.7)
**Construction**,** architecture**,** surveying and building technology**	**394**	**21**	**5.6 (3.6–8.6)**	**286**	**23**	**9.7 (6.6–14.1)**	**1.4 (1.0**–**2.1)**
Construction planning, architecture, surveying	131	5	4.7 (2.0–10.4)	100	5	6.2 (2.6–13.5)	0.9 (0.4–2.0)
Building and supply services	150	8	5.3 (2.4–10.6)	105	12	12.9 (7.3–21.4)	1.9 (1.1–3.1)
**Natural Science**,** geography and computer science**	**549**	**19**	**3.9 (2.4–6.1)**	**435**	**30**	**7.5 (5.1–10.7)**	**1.1 (0.7–1.6)**
Mathematics, biology, chemistry, physics	156	5	2.8 (0.9–7.3)	121	11	12.1 (7.0–20.0)	1.8 (1.0–2.9)
Computer science, information and communication technologies	369	12	3.8 (2.1–6.7)	299	19	5.9 (3.5–9.7)	0.8 (0.5–1.4)
**Transport**,** logistics**,** protection and safety**	**716**	**50**	**8.3 (6.4–10.8)**	**448**	**44**	**9.5 (6.9–12.9)**	**1.4 (1.0–1.9)**
Traffic, logistics (except vehicle driver)	288	19	7.0 (4.3–10.9)	172	12	7.3 (3.9–12.7)	1.1 (0.6–1.8)
Drivers and mobile plant operators	204	20	12.0 (7.9–17.7)	128	20	14.8 (9.0–22.9)	2.2 (1.3–3.3)
Protection, security and surveillance	150	5	4.6 (1.9–10.0)	111	5	4.5 (1.6–11.2)	0.7 (0.2–1.6)
Cleaners	74	37	10.5 (5.2–19.8)	37	7	16.5 (7.1–32.6)	2.2 (1.0–4.7)
**Commercial services**,** trade in goods**,** sales**,** hotel and tourism**	**801**	**46**	**7.2 (5.6–9.2)**	**578**	**39**	**7.7 (5.8–10.2)**	**1.1 (0.8–1.5)**
Purchasing, sales and distribution, trade	279	16	6.4 (3.9–10.2)	220	9	5.2 (2.7–9.3)	0.8 (0.4–1.4)
Sales workers (retail)	377	24	8.4 (6.0–11.6)	250	18	7.9 (5.1–11.9)	1.1 (0.8–1.7)
Tourism, hotel and restaurant	145	6	5.2 (2.5–10.2)	108	12	12.0 (7.0–19.7)	1.7 (1.0–2.9)
**Business organisation**,** accounting**,** law and public administration**	**2401**	**88**	**4.5 (3.8–5.4)**	**1799**	**119**	**7.1 (5.9–8.4)**	**1.0 (0.9–1.2)**
Corporate management and business organisation	1257	41	3.9 (3.0–5.2)	951	57	6.7 (5.2–8.5)	1.0 (0.8–1.2)
Financial services, accounting, tax consultancy	575	24	4.9 (3.3–7.1)	440	29	6.6 (4.5–9.4)	0.9 (0.7–1.4)
Law and administration	569	23	5.5 (3.8–7.7)	408	33	8.6 (6.1–11.8)	1.2 (0.9–1.7)
**Health**,** social affairs**,** teaching and education**	**1553**	**52**	**3.7 (2.9**–**4.8)**	**1161**	**47**	**4.0 (3.0–5.3)**	**0.6 (0.4–0.8)**
Medical health professions	616	14	2.3 (1.3–3.8)	464	11	2.5 (1.4–4.4)	0.4 (0.2–0.7)
Non-medical health professions, personal care, medical technology	162	6	4.3 (2.0–8.9)	126	6	4.6 (1.9–10.2)	0.7 (0.3–1.5)
Education, social and domestic professions, theology	318	13	4.3 (2.5–7.3)	233	14	6.0 (3.5–10.1)	0.9 (0.5–1.5)
Teaching professions	457	19	5.0 (3.4–7.4)	338	16	4.4 (2.7–7.2)	0.7 (0.4–1.0)
**Humanities**,** culture**,** design**	**392**	**12**	**3.9 (2.3–6.6)**	**302**	**14**	**5.1 (2.9–8.6)**	**0.7 (0.4–1.3)**
Advertising, marketing, commercial and editorial media professions	249	8	4.9 (2.6–8.7)	191	10	5.4 (2.7–10.1)	0.8 (0.4–1.5)
**Job complexity**							
Low	292	16	6.3 (4.0–9.7)	161	16	10.6 (6.7–16.3)	1.5 (1.0–2.4)
Medium	3629	192	6.2 (5.4–7.0)	2549	201	8.4 (7.4–9.6)	1.2 (1.1–1.4)
High	1705	76	4.9 (3.9–6.1)	1291	76	5.8 (4.6–7.3)	0.8 (0.7–1.1)
Very high	2504	81	4.0 (3.3–4.9)	1953	95	5.2 (4.3–6.3)	0.8 (0.6–0.9)
**Management position**							
Supervisors and manager	975	37	4.8 (3.6–6.4)	764	51	7.3 (5.6–9.5)	1.1 (0.8–1.4)
No managerial position	7155	328	5.3 (4.8–5.9)	5190	337	6.8 (6.2–7.6)	1.0 (0.9–1.1)

Furthermore, a dose-response relationship was seen between job complexity level and incidence of type 2 diabetes. The highest incidence was observed among employees who worked in jobs with low job complexity level (10.6%) and decreased as the level of complexity increased. Employees in jobs with low complexity level had a 50% higher incidence of type 2 diabetes (SIR = 1.5, 95% CI 1.0–2.4). The incidence among supervisors and managers was similar compared to employees without a managerial position (7.3% and 6.8%) (Table 2).

In men, the incidence of the main occupational group of “food production and processing” (SIR = 3.0, 95% CI 1.4–5.6) was increased threefold and more than twofold in “tourism, hotel and restaurant” (SIR = 2.4, 95% CI 0.9–5.0) and “drivers and mobile plant operators” (SIR = 2.3, 95% CI 1.4–3.5). Female employees had the highest SIR in “mathematics, biology, chemistry, physics” (SIR = 2.8, 95% CI 1.3–4.7) and in “cleaners” (SIR = 2.1, 95% CI 0.7–4.4). The sex-stratified incidence rates and SIR of type 2 diabetes are in the supplementary material (see additional file 3).

### Incidence of metabolic syndrome

**Fig. 3:**
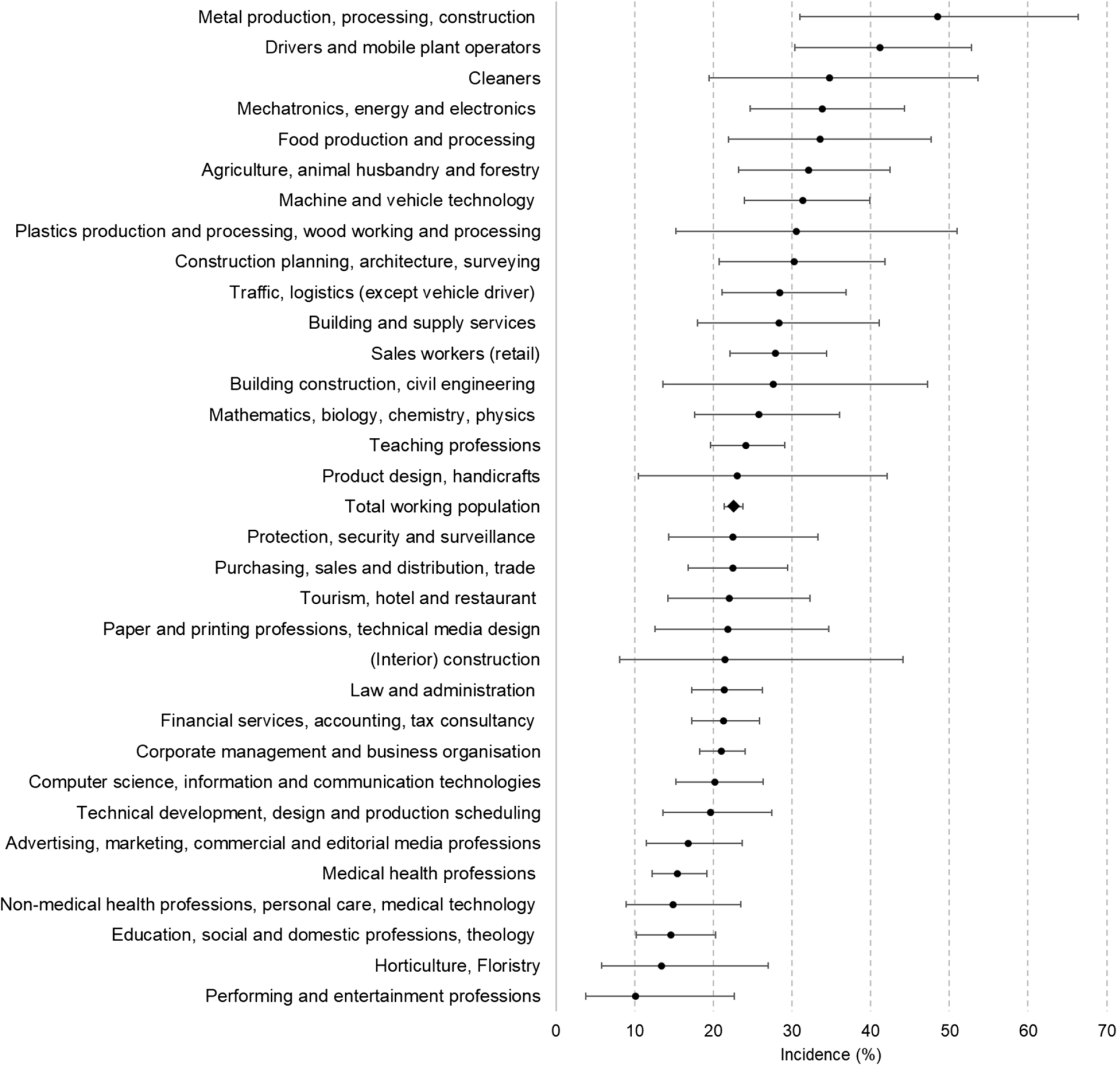
Age- and sex-standardized incidence and 95% CI of metabolic syndrome, stratified by main occupational group

Within the 10-year observation period, 1104 participants of the working population developed metabolic syndrome, and age- and sex-standardized incidence was 22.6%. The highest 10-year incidence was observed in the occupational area of “transport, logistics, protection and safety” (30.6%). Comparison of the main occupational groups showed the highest incidence among employees in “metal production, processing and construction” (48.5%), “drivers and mobile plant operators” (41.2%), and “cleaners” (34.8%). The lowest incidence of metabolic syndrome was seen in “performing and entertainment professions” (10.1%) and “horticulture and floristry” (13.4%) (Fig. 3; Table 3).

**Table 3:** Age- and sex-standardised incidence and SIR of metabolic syndrome for occupational areas and main occupational groups, job complexity level and managerial position

	*N* Baseline	Prevalent cases	Age- and sex-standardised Prevalence (95% CI)	Persons at risk	Incident cases	Age- and sex-standardized Incidence (95% CI)	SIR (95% CI)
**Total working population**	**8153**	**1736**	**22.9 (22.0–23.8)**	**5103**	**1104**	**22.6 (21.4–23.8)**	**–**
**Agriculture, forestry, animal husbandry and horticulture**	**229**	**46**	**20.6 (15.8–26.4)**	**144**	**36**	**25.9 (19.1–34.0)**	**1.1 (0.9–1.5)**
Agriculture, animal husbandry and forestry	151	34	23.9 (17.7–31.2)	94	29	32.1 (23.2–42.5)	1.4 (1.0–1.9)
Horticulture, Floristry	78	12	13.5 (7.0–23.7)	50	7	13.4 (5.8–27.0)	0.6 (0.3–1.2)
**Raw material extraction**,** production and manufacturing**	**1097**	**289**	**27.9 (25.2–30.8)**	**649**	**179**	**28.7 (25.0–32.6)**	**1.3 (1.1–1.4)**
Plastics production and processing, wood working and processing	54	14	28.6 (17.0–43.6)	31	8	30.5 (15.2–51.0)	1.1 (0.7–2.3)
Paper and printing professions, technical media design	111	25	25.0 (17.2–34.8)	69	15	21.8 (12.6–34.7)	1.0 (0.6–1.5)
Metal production, processing and construction	76	28	40.5 (29.1–53.1)	38	17	48.5 (31.0–66.4)	2.1 (1.4–2.9)
Machine and vehicle technology	269	67	26.1 (20.7–32.2)	162	49	31.4 (23.9–39.9)	1.4 (1.1–1.8)
Mechatronics, energy and electronics	188	61	34.0 (27.0–41.7)	110	37	33.8 (24.7–44.3)	1.5 (1.1–2.0)
Technical development, design and production scheduling	253	58	23.4 (18.2–29.6)	160	31	19.6 (13.6–27.4)	0.9 (0.6–1.2)
Food production and processing	99	26	27.7 (19.6–37.5)	53	16	33.6 (21.9–47.7)	1.5 (1.0–2.1)
**Construction**,** architecture**,** surveying and building technology**	**394**	**118**	**32.3 (27.6–37.3)**	**217**	**59**	**28.2 (22.1–35.1)**	**1.2 (1.0–1.6)**
Construction planning, architecture, surveying	131	33	26.7 (19.4–35.3)	81	22	30.3 (20.7–41.8)	1.3 (0.9–1.9)
Building construction, civil engineering	57	14	27.6 (16.5–42.1)	36	10	27.6 (13.6–47.2)	1.2 (0.6–2.1)
(Interior) construction	56	13	22.6 (12.0–37.8)	30	7	21.5 (8.1–44.1)	1.0 (0.4–2.0)
Building and supply services	150	58	41.3 (33.4–49.7)	70	20	28.3 (18.0–41.1)	1.2 (0.8–1.8)
**Natural Science**,** geography and computer science**	**552**	**122**	**22.9 (19.3–26.9)**	**368**	**82**	**22.5 (18.2–27.6)**	**1.0 (0.8–1.2)**
Mathematics, biology, chemistry, physics	158	40	25.1 (18.5–33.0)	102	24	25.8 (17.6–36.0)	1.1 (0.8–1.6)
Computer science, information and communication technologies	370	79	22.2 (17.8–27.2)	251	53	20.2 (15.2–26.3)	0.9 (0.7–1.2)
**Transport**,** logistics**,** protection and safety**	**719**	**209**	**30.8 (27.4–34.5)**	**359**	**110**	**30.6 (25.7–36.0)**	**1.4 (1.1–1.6)**
Traffic, logistics (except vehicle driver)	289	76	26.2 (21.1–31.9)	143	43	28.4 (21.1–36.9)	1.3 (0.9–1.6)
Drivers and mobile plant operators	205	80	42.2 (35.2–49.6)	93	37	41.2 (30.4–52.8)	1.9 (1.3–2.3)
Protection, security and surveillance	150	36	26.0 (19.1–34.4)	92	20	22.5 (14.3–33.3)	1.0 (0.6–1.5)
Cleaners	75	17	27.5 (18.5–38.5)	31	10	34.8 (19.5–53.6)	1.5 (0.9–2.4)
**Commercial services**,** trade in goods**,** sales**,** hotel and tourism**	**806**	**175**	**24.6 (21.8–27.7)**	**488**	**103**	**24.8 (21.1–28.9)**	**1.1 (0.9–1.3)**
Purchasing, sales and distribution, trade	280	57	21.2 (16.6–26.6)	190	37	22.5 (16.8–29.4)	1.0 (0.7–1.3)
Sales workers (retail)	380	88	26.6 (22.5–31.2)	209	48	27.9 (22.1–34.4)	1.2 (1.0–1.5)
Tourism, hotel and restaurant	146	30	25.0 (18.7–32.5)	89	18	22.0 (14.2–32.3)	1.0 (0.6–1.4)
**Business organisation**,** accounting**,** law and public administration**	**2404**	**480**	**21.8 (20.2–23.5)**	**1559**	**315**	**21.2 (19.2–23.3)**	**0.9 (0.9–1.0**)
Corporate management and business organisation	1259	233	20.2 (18.1–22.5)	831	166	21.0 (18.3–24.0)	0.9 (0.8–1.1)
Financial services, accounting, tax consultancy	575	114	20.8 (17.6–24.4)	382	82	21.3 (17.3–25.9)	0.9 (0.8–1.2)
Law and administration	570	133	26.1 (22.7–29.9)	346	67	21.4 (17.3–26.2)	1.0 (0.8–1.2)
**Health**,** social affairs**,** teaching and Education**	**1558**	**244**	**17.3 (15.5–19.2)**	**1041**	**173**	**17.8 (15.6–20.3)**	**0.8 (0.7–0.9)**
Medical health professions	618	75	14.0 (11.5–17.0)	431	70	15.4 (12.2–19.2)	0.7 (0.5–0.9)
Non-medical health professions, personal care, medical technology	162	37	23.0 (17.0–30.2)	103	15	14.9 (8.9–23.5)	0.7 (0.4–1.0)
Education, social and domestic professions, theology	319	56	18.0 (14.1–22.6)	204	28	14.6 (10.2–20.3)	0.6 (0.5–0.9)
Teaching professions	459	76	19.0 (15.8–22.7)	303	60	24.1 (19.6–29.1)	1.1 (0.9–1.3)
**Humanities**,** culture**,** design**	**394**	**53**	**16.0 (12.5–20.2)**	**278**	**47**	**17.0 (12.7–22.4)**	**0.8 (0.6–1.0)**
Advertising, marketing, commercial and editorial media professions	249	34	16.3 (11.9–21.8)	176	29	16.8 (11.5–23.7)	0.8 (0.5–1.1)
Product design, handicrafts	43	5	12.1 (4.9–25.8)	29	7	23.0 (10.5–42.1)	1.0 (0.5–1.9)
Performing and entertainment professions	83	12	16.5 (9.3–27.2)	59	6	10.1 (3.8–22.7)	0.4 (0.2–1.0)
**Job complexity**							
Low	294	74	26.4 (21.7–31.6)	139	36	27.0 (20.3–34.8)	1.2 (0.9–1.5)
Medium	3640	808	24.0 (22.6–25.4)	2180	505	24.0 (22.2–25.8)	1.1 (1.0–1.1)
High	1709	383	23.3 (21.3–25.4)	1079	231	21.8 (19.3–24.5)	1.0 (0.9–1.1)
Very high	2510	471	20.7 (19.1–22.3)	1705	332	20.9 (19.0–23.0)	0.9 (0.8–1.0)
**Management position**							
Supervisors and manager	976	249	27.3 (24.5–30.3)	609	142	25.0 (21.6–28.8)	1.1 (1.0–1.3)
No managerial position	7177	1487	22.3 (21.4–23.3)	4494	962	22.3 (21.1–23.6)	1.0 (0.9–1.0)

SIR calculations showed a two-fold increased incidence for employees in “metal production, processing, and construction” (SIR = 2.1, 95% CI 1.4–2.9) and for “drivers and mobile plant operators” (SIR = 1.9, 95% CI 1.3–2.3), and a 50% higher incidence for “cleaners” (SIR = 1.5, 95% CI 0.9–2.4) as well as employees in “mechatronics, energy and electronics” (SIR = 1.5, 95% CI 1.1–2.0) and “food production and processing” (SIR = 1.5, 95% CI 1.0–2.1) (Table 3).

Again, an increased incidence was observed with decreasing job complexity levels. The incidence of metabolic syndrome in employees with low job complexity level was 27.0%, and compared to the total working population, the incidence was 20% higher (SIR = 1.2; 95% CI 0.9–1.5).

Supervisors and managers had a slightly higher incidence of metabolic syndrome than participants without a managerial position (25.0% vs. 22.3%, respectively) (Table 3).

The highest SIR for men was observed in “metal production, processing and construction” (SIR = 2.2, 95% CI 1.5–3.1) and for women in “cleaners” (SIR = 1.5, 95% CI 0.8–2.4) and in “mathematics, biology, chemistry, physics” (SIR = 1.5, 95% CI 0.9–2.2). A complete overview of the sex-stratified incidence rates and SIR of metabolic syndrome can be found in the supplementary material (see additional file 4).

## Discussion

### Principal findings and comparison to other studies

In the present study, we found wide differences in the incidence of type 2 diabetes and metabolic syndrome between occupational groups and identified occupations with increased incidences. The highest incidence of type 2 diabetes was found in “food production and processing” and for metabolic syndrome in “metal production, processing and construction”. There was also a high incidence of both type 2 diabetes and metabolic syndrome in “cleaners” and “drivers and mobile plant operators”, and several other occupational groups also showed a distinct higher incidence compared to the total working population.

A study of German health insurance records showed that the occupational areas “transport, logistics, protection and security” and “health sector, social work, teaching and education” had the highest predicted probabilities for type 2 diabetes [18]. The results of this study only partially agree with ours, as we also observed increased incidences in “transport, logistics, protection and safety”. However, the occupational area “health, social affairs, teaching and education” had the lowest incidence in our study.

Previous cross-sectional studies showed similar occupational groups with high prevalence of type 2 diabetes or metabolic syndrome. In a US study, the highest age-adjusted prevalence of metabolic syndrome was found in “food preparation and food service workers” and “farm operators, managers, and supervisors” (29.6–31.1%) [12]. In Japan, metabolic syndrome was most prevalent among males working in “construction”, “transportation”, “professional services”, and “cooperative sectors” and among females working in the “health care sector” and “cooperative associations” [11]. A New Zealand study showed similar results, which found an increased risk of type 2 diabetes among “plant and machine operators” and “assemblers” [26].

Our results are also largely consistent with existing longitudinal studies. A nationwide Swedish register-based study indicates that “professional drivers”, “manufacturing workers,” and “cleaners” have a threefold increased risk of type 2 diabetes compared to occupations in “health and education” sectors [16]. Similarly, a study of 75,000 Dutch workers showed an increased 3.8-year incidence of metabolic syndrome in male “stationary plant and machine operators”, “electrical and electronics trades workers,” and female “food preparation assistants” and “drivers and mobile plant operators” [17].

Interestingly, the occupational groups with high incidences are very heterogeneous in terms of their occupational exposures. Working conditions such as (night) shift work [27], long working hours [28], job strain [29] and high sedentary time [30] have been associated with an increased risk for metabolic disorders.

Furthermore, most of the occupational groups with a high incidence can be categorized as elementary and manual occupations. Previous studies have shown that “blue-collar workers”, who are predominantly engaged in manual labour, have a higher risk for type 2 diabetes and hypertension compared to “white-collar workers” [31, 32].

However, the SIR in this study cannot be interpreted as a purely occupational risk. The results may also reflect the socioeconomic inequality that exists across the different occupational groups. One indication is the dose-response relationship we observed between decreasing job complexity and an increased incidence of type 2 diabetes and metabolic syndrome. Employees with low or medium level of job complexity in our study are likely to work in predominantly “blue-collar” jobs, while those with high and very high levels of job complexity are more likely to work in “white-collar” jobs. Typically, “blue-collar workers” are often characterized by a low SES with a low educational attainment, which is in turn associated with type 2 diabetes [33, 34]. This assumption is supported by the study of Carlsson et al. [16], which showed that occupations with the highest incidence of type 2 diabetes were those characterized by low SES. In addition, a cohort study showed that low-skilled workers, especially in blue-collar occupations, had a significantly higher risk of metabolic syndrome than high-skilled white-collar workers and were more likely to exhibit unhealthy behaviour such as smoking, unhealthy diet, and low leisure time physical activity [35]. Therefore, occupation may indicate the presence of cardiometabolic risk factors. On the other hand, there are also major differences about the cardiometabolic risk profile in white-collar occupations, regardless of SES, which can be attributed to the heterogeneity in the respective occupations [26].

Occupational factors and exposures may also be contributing to risk. The longitudinal analysis by van Zon et al. shows that half of the corresponding occupational groups still had an increased risk of metabolic syndrome after adjustment for age and health behaviour [17]. Further, white-collar occupations showed significantly higher cholesterol and stress levels [31]. This supports the hypothesis that the occupational group plays an important role in the development of metabolic syndrome and does not just reflect the selection of workers with less healthy lifestyles into certain occupations.

The risk for type 2 diabetes and metabolic syndrome is likely the accumulated effects of SES, lifestyle factors, and occupational exposures. The partially conflicting results reflect the complexity of these relationships and more research is needed to investigate the extent to which these factors influence the occurrence of type 2 diabetes and metabolic syndrome. Our results primarily show which occupational groups are particularly vulnerable and would potentially benefit most from preventive measures.

### Strengths and limitations of the study

To our knowledge, this is the first study that examined both type 2 diabetes and metabolic syndrome across common occupational groups. A main strength of the study is its long follow-up period of 10 years. This is particularly important because type 2 diabetes develops gradually and is often only diagnosed after a long latency period. Also, recruiting a large population-based study sample means that the study included a wide range of occupations. The occupations were coded twice by two coders for 10% of the subjects independently. A further strength is that the results are based on objectively measured outcomes taken by trained personnel and medication use was taken into account. This means that undiagnosed cases of type 2 diabetes and metabolic syndrome were considered. This helps avoid underestimation of incidence because there is a high estimated number of unreported cases. In 2021, globally almost every second adult (20–79 years old) with type 2 diabetes was unaware of their diabetes status [36]. In Europe, the proportion of undiagnosed cases was 35.7%.

Further, we tried to ensure comparability with other studies regarding metabolic syndrome by using the harmonised definition of Alberti et al. [23], which attempts to unify the different criteria of the different organisations.

Our study has some limitations. A limiting fact is the small number of individuals in some occupational groups, resulting in few observed cases and wide CIs. The SIRs in these groups should therefore be interpreted and discussed with caution, because any risk difference is questionable if the CI encompasses the one. However, we counteracted this by only examining occupations with at least 20 people in the occupational group. Although our goal was to provide representative results, workers with low job complexity were difficult to recruit and are therefore underrepresented in this study. Standardisation according to SES or job complexity should possibly be considered in future studies.

Also, certain occupational groups are still dominated by women or men, making the comparison of the sex-stratified results challenging. Previous studies have also found sex differences in terms of incidence in the same occupational groups, but these differences were predominantly found in female- or male-dominated occupations [16, 17]. We also found sex-specific differences in occupations with an even distribution of women and men, such as the main occupational groups of the area “business organisation, accounting, law and public administration”.

Furthermore, we cannot rule out selection bias, as a proportion of participants did not participate in the 10-year follow-up. Within the 10 years, only about 5% of the included participants changed the occupational area and 7% the main occupational group, but we cannot rule out bias due to a change of occupation during the observation period.

Although we standardised for age and sex to ensure national comparability, it is unclear to what extent our results can be transferred to other regions in Germany with different socioeconomic structures or other countries with different health care systems. Further, the retention rate at the 10-year follow-up was 63.8%, acceptable for such a long follow-up period. However, lost to follow-up analyses have shown that employees with an unhealthy lifestyle, low job complexity and from the occupational area of “transport, logistics, protection and safety” have a higher probability not to participate at the follow-up (unpublished analyses).

### Meaning of the study and unanswered questions

The aim of this study was to investigate the incidence of type 2 diabetes and metabolic syndrome of a working population in Germany and to identify occupational groups with an increased risk over a period of 10 years. Alarmingly, we found that in some occupational groups, almost half of the employees met the criteria for metabolic syndrome and that in some occupational groups, the incidence of metabolic syndrome and type 2 diabetes was two to three times higher compared to the total working population in this study. Thus, our study illustrates the high need for preventive measures at the workplace, particularly in vulnerable occupations like “food production and processing”, “metal production, processing and construction”, “cleaners”, and “drivers, and mobile plant operators”, but also in further occupational groups. Our results also point to the heterogeneity of the incidences of the individual occupational main groups within an occupational area, meaning occupational areas with a high incidence also include occupational groups with low incidence, and vice versa. Therefore, occupations should be investigated and addressed as differentiated as possible for future studies and prevention initiatives.

Particularly in the face of demographic change, increasing retirement age and an increasing shortage of skilled workers, prevention is essential in all areas of life to ensure a healthy workforce. As most employees spend a large part of their time at work and are exposed to potential risk factors there, workplace prevention initiatives represent an important approach. Previous studies have provided evidence that preventive measures in the workplace could be useful and effective in reducing risk factors associated with type 2 diabetes [37]. In addition to the prevention of type 2 diabetes and metabolic syndrome, their progression and complications can be prevented when diagnosed. Thus, our results provide guidance, not only for primary and secondary prevention (incidence), but also for tertiary prevention (prevalence).

The occupations with high incidence identified in this study provide indications of where health promotion and screening programs as part of occupational health check-ups could bring the greatest benefit. In addition, workplace adjustments for people with existing cardiometabolic risk factors may be necessary to avoid complications and comorbidities.

Our study findings have implications for further research. First, vulnerable occupational groups and their work-related risk factors should be examined in more detail to be able to implement targeted and successful preventive measures. Second, future studies should investigate the complex relationship between occupational exposure, SES, lifestyle factors, and cardiometabolic risk. Third, it should be examined to what extent preventive measures in the workplace especially in occupations with increased risk can be made more effective in promoting health and preventing cardiometabolic diseases.

## Conclusions

In conclusion, this study expands the existing evidence on the incidence of type 2 diabetes and metabolic syndrome in different occupational groups and highlights the vulnerability of certain occupations such as “food production and processing”, “metal production, processing and construction”, “cleaners” and “drivers and mobile plant operators”.

Our findings are consistent with previous research indicating increased health risk in these and similar occupations and underline the urgent need for targeted preventive measures within these work environments. As occupations serve not only as indicators of cardiometabolic risk but also as platforms for intervention, the findings of this study could guide the development of more nuanced and effective workplace health initiatives. Moving forward, it is imperative to address these occupational health disparities through comprehensive and tailored strategies to reduce the risks associated with type 2 diabetes and metabolic syndrome, and ultimately promote a healthier workforce for the future.

## Supplementary Information

Supplementary Material 1.

Supplementary Material 2.

Supplementary Material 3.

Supplementary Material 4.

## Data Availability

The data that support the findings of this study are available from the Gutenberg Health Study (GHS), but restrictions apply to the availability of these data, which were used under licence for the current study and so are not publicly available. Data are however available from the authors upon reasonable request and with permission of GHS.

## Abbreviations

ATC: Anatomical therapeutic chemical
CI: Confidence Interval
GHS: Gutenberg Health Study
HDL: High-density lipoprotein cholesterol
KldB: Klassifikation der Berufe (classification of occupations)
SES: Socioeconomic Status
SIR: Standardised incidence ratio
