# Supplementary material for: Incidence of type 2 diabetes and metabolic syndrome by Occupation – 10-Year follow-up of the Gutenberg Health Study
Source: BMC Public Health. 2025 Feb 7;25:502. doi: 10.1186/s12889-025-21732-5 (PMC11803924; doi:10.1186/s12889-025-21732-5)
Supplement: Supplementary file 1 — Supplementary Material 1. [file 12889_2025_21732_MOESM1_ESM.docx]

**Additional file 2:**

**Incidence of Type 2 Diabetes and Metabolic Syndrome by Occupation – 10-Year Follow-up of the Gutenberg Health Study**

Weighting factors for age- and sex-standardisation

| **Age groups [5y]** | **Men** | **Women** |
| --- | --- | --- |
| 35-39 | 0.91762175 | 1.10980435 |
| 40-44 | 0.79110008 | 0.83440047 |
| 45-49 | 0.62277416 | 0.73145168 |
| 50-54 | 0.8500006 | 0.94017856 |
| 55-59 | 1.03447745 | 1.3841587 |
| 60-64 | 1.71603325 | 2.90552585 |
