# Supplementary material for: Incidence of type 2 diabetes and metabolic syndrome by Occupation – 10-Year follow-up of the Gutenberg Health Study
Source: BMC Public Health. 2025 Feb 7;25:502. doi: 10.1186/s12889-025-21732-5 (PMC11803924; doi:10.1186/s12889-025-21732-5)
Supplement: Supplementary file 2 — Supplementary Material 2. [file 12889_2025_21732_MOESM2_ESM.docx]

**Additional file 1:**

**Incidence of Type 2 Diabetes and Metabolic Syndrome by Occupation – 10-Year Follow-up of the Gutenberg Health Study**

**Questionnaire**

| **Educational and vocational qualifications** | |
| --- | --- |
| What is the highest educational qualification you have?  What other qualification do you have? | 1 = Secondary general school  2 = Intermediate school  3 = High school diploma  4 = Other educational qualification  5 = No qualification |
| What is the highest vocational qualification you have?  What other qualification do you have? | 1 = Primary vocational school  2 = Secondary vocational school  3 = University degree  4 = Other professional qualification  5 = No qualification |
| **Occupational phases** | |
| Are you currently employed? | 0 = No  1 = Yes, full-time  2 = Yes, regularly employed part-time  3 = Yes, marginally employed or irregularly employed |
| What is your current job? | 100 characters |
| How long have you been working in this job? | yyyy |
| Please describe your job. | 100 characters |
| What industry does the company belong to? | 100 characters |
| Please state below how many hours you worked on average per week: first the fixed working hours and then the number of overtime hours.  How many hours do you work on average per week in fixed working hours?  How many overtime hours do you work on average per week? | ____ hours per week  ____ hours per week |
| Were there any other career phases apart from the current one? If "yes", these will be asked chronologically, starting with your first job or training. | 0 = no  1 = yes |
| What was your first job after school that you did for at least 12 months? | 100 characters |
| 1st occupational period: start of career  1st occupational period: end of job | mm.yyyy  mm.yyyy |
| Please describe your job. | 100 characters |
| What industry does the company belong to? | 100 characters |
| Please state below how many hours you worked on average per week: first the fixed working hours and then the number of overtime hours.  How many hours did you work on average per week in fixed working hours?  How many overtime hours did you work on average per week? | ____ hours  ____ hours |
| What was your 2nd job after school that you did for at least 12 months? | 100 characters |
| *[Repetition of the questions about the occupational periods; up to 15 occupational phases possible]* | |
| **Diabetes** | |
| Do you have diabetes? | 0 = no  1 = yes  99 = don't know |
| Has your diabetes been diagnosed by a physician? | 0 = no  1 = yes  99 = don't know |
| What type of diabetes do you have? | 1 = “Juvenile diabetes” (type 1)  2 = “Adult-onset diabetes” (type 2)  3 = Gestational diabetes  4 = Diabetes after pancreatitis  99 = Don’t know |
| Please indicate the year in which you were diagnosed with diabetes. | yyyy |
| Is your diabetes currently being treated with a diet, tablets, insulin or a combination of tablets and insulin? | 1 = Only with tablets  2 = Only with insulin  3 = With insulin and tablets  4 = Only diet  5 = No treatment |
