# Supplementary material for: Incidence of type 2 diabetes and metabolic syndrome by Occupation – 10-Year follow-up of the Gutenberg Health Study
Source: BMC Public Health. 2025 Feb 7;25:502. doi: 10.1186/s12889-025-21732-5 (PMC11803924; doi:10.1186/s12889-025-21732-5)
Supplement: Supplementary file 3 — Supplementary Material 3. [file 12889_2025_21732_MOESM3_ESM.docx]

**Additional file 4:**

**Incidence of Type 2 Diabetes and Metabolic Syndrome by Occupation – 10-Year Follow-up of the Gutenberg Health Study**

Age- and sex-standardised incidence and SIR of metabolic syndrome for occupational areas and main occupational groups, stratified by sex

|  | **Men** | | | | | **Women** | | | | |
| --- | --- | --- | --- | --- | --- | --- | --- | --- | --- | --- |
| **Occupation** | **N** | **Persons at risk** | **Incident cases** | **Age- and sex-standardised Incidence**  **(95% CI)** | **SIR (95% CI)** | **N** | **Persons at risk** | **Incident cases** | **Age- and sex-standardised Incidence**  **(95% CI)** | **SIR (95% CI)** |
| **Agriculture, forestry, animal husbandry and horticulture** | **170** | **102** | **27** | **27.5 (19.2–37.8)** | **1.2 (0.9–1.7)** | **59** | **42** | **9** | **22.6 (12.3–37.4)** | **1.0 (0.5–1.7)** |
| Agriculture, animal husbandry and forestry | 111 | 67 | 22 | 33.9 (22.8–46.9) | 1.5 (1.0–2.1) | 40 | 27 | 7 | 28.6 (14.8–47.5) | 1.2 (0.7–2.1) |
| Horticulture, Floristry | 59 | 35 | 5 | 14.8 (5.4–32.6) | 0.7 (0.2–1.4) | – | | | | |
| **Raw material extraction, production and manufacturing** | **931** | **546** | **162** | **31.1 (27.0–35.6)** | **1.4 (1.2–1.6)** | **166** | **103** | **17** | **18.0 (11.5–26.8)** | **0.8 (0.5–1.2)** |
| Plastics production and processing, wood working and processing | 51 | 30 | 8 | 31.6 (15.8–52.5) | 1.4 (0.7–2.3) | – | | | | |
| Paper and printing professions, technical media design | 69 | 39 | 12 | 33.7 (18.4–53.0) | 1.4 (0.8–2.3) | – | | | | |
| Metal production, processing and construction | 74 | 36 | 17 | 51.6 (33.1–69.7) | 2.2 (1.5–3.1) | – | | | | |
| Machine and vehicle technology | 258 | 154 | 47 | 31.6 (23.9–40.4) | 1.4 (1.1–1.8) | – | | | | |
| Mechatronics, energy and electronics | 186 | 108 | 37 | 34.5 (25.2–45.0) | 1.5 (1.1–2.0) | – | | | | |
| Technical development, design and production scheduling | 207 | 135 | 27 | 20.4 (13.7–29.2) | 0.9 (0.6–1.3) | – | | | | |
| Food production and processing | 55 | 28 | 11 | 43.1 (24.4–63.8) | 2.0 (1.1–2.8) | 44 | 25 | 5 | 25.6 (12.3–45.1) | 1.2 (0.5–2.0) |
| **Construction, architecture, surveying and building technology** | **366** | **200** | **58** | **30.5 (23.9–37.9)** | **1.4 (1.06–1.68)** | **–** | | | | |
| Construction planning, architecture, surveying | 109 | 66 | 21 | 36.3 (24.8–49.4) | 1.6 (1.1–2.2) | – | | | | |
| Building construction, civil engineering | 56 | 35 | 10 | 28.5 (14.1–48.5) | 1.2 (0.6–2.2) | – | | | | |
| (Interior) construction | 56 | 30 | 7 | 21.5 (8.1–44.1) | 1.0 (0.4–2.0) | – | | | | |
| Building and supply services | 145 | 69 | 20 | 28.8 (18.4–41.8) | 1.3 (0.8–1.9) | – | | | | |
| **Natural Science, geography and computer science** | **445** | **282** | **63** | **22.2 (17.2–28.1)** | **1.0 (0.8–1.2)** | **107** | **86** | **19** | **23.5 (15.2–34.2)** | **1.1 (0.7–1.5)** |
| Mathematics, biology, chemistry, physics | 101 | 56 | 10 | 18.1 (9.1–32.1) | 0.8 (0.4–1.4) | 57 | 46 | 14 | 34.2 (21.2–49.9) | 1.5 (0.9–2.2) |
| Computer science, information and communications technologies | 328 | 217 | 50 | 22.5 (16.8–29.4) | 1.0 (0.7–1.3) | – | | | | |
| **Transport, logistics, protection and safety** | **534** | **255** | **93** | **37.1 (30.8–43.9)** | **1.7 (1.4–1.9)** | **185** | **104** | **17** | **17.3 (10.9–26.1)** | **0.8 (0.5–1.2)** |
| Traffic, logistics (except vehicle driver) | 205 | 94 | 38 | 39.9 (29.5–51.4) | 1.8 (1.3–2.3) | 84 | 49 | 5 | 10.1 (3.9–22.4) | 0.4 (0.2–1.0) |
| Drivers and mobile plant operators | 195 | 87 | 35 | 41.6 (30.4–53.6) | 1.9 (1.3–2.4) | – | | | | |
| Protection, security and surveillance | 123 | 71 | 19 | 28.2 (17.9–41.2) | 1.3 (0.8–1.8) | – | | | | |
| Cleaners | – | | | | | 64 | 28 | 9 | 34.6 (18.9–54.2) | 1.5 (0.8–2.4) |
| **Commercial services, trade in goods, sales, hotel and tourism** | **353** | **217** | **49** | **24.1 (18.4–30.8)** | **1.1 (0.8–1.4)** | **453** | **271** | **53** | **25.3 (20.5–30.7)** | **1.1 (0.9–1.4)** |
| Purchasing, sales and distribution, trade | 197 | 130 | 29 | 25.2 (17.8–34.1) | 1.1 (0.8–1.5) | 83 | 60 | 8 | 17.5 (9.5–29.5) | 0.8 (0.4–1.3) |
| Sales workers (retail) | 111 | 64 | 16 | 24.6 (14.6–38.0) | 1.1 (0.7–1.7) | 269 | 145 | 32 | 29.0 (22.3–36.8) | 1.3 (1.0–1.6) |
| Tourism, hotel and restaurant | – | | | | | 101 | 66 | 14 | 23.6 (14.4–35.9) | 1.1 (0.6–1.6) |
| **Business organisation, accounting, law and public administration** | **1008** | **602** | **165** | **28.2 (24.4–32.2)** | **1.2 (1.1–1.4)** | **1396** | **957** | **150** | **17.6 (15.3–20.1)** | **0.8 (0.7–0.9)** |
| Corporate management and business organisation | 497 | 304 | 90 | 30.0 (24.6–35.9) | 1.3 (1.1–1.6) | 762 | 527 | 76 | 16.8 (13.9–20.2) | 0.8 (0.6–0.9) |
| Financial services, accounting, tax consultancy | 274 | 174 | 47 | 28.0 (21.2–36.0) | 1.2 (1.0–1.6) | 301 | 208 | 35 | 16.8 (12.2–22.4) | 0.7 (0.5–1.0) |
| Law and administration | 237 | 124 | 28 | 24.2 (16.8–33.3) | 1.1 (0.7–1.5) | 333 | 222 | 39 | 20.1 (15.3–25.9) | 0.9 (0.7–1.2) |
| **Health, social affairs, teaching and Education** | **458** | **281** | **67** | **25.0 (20.0–30.9)** | **1.1 (0.9–1.4)** | **1100** | **760** | **106** | **15.6 (13.2–18.3)** | **0.7 (0.6–0.8)** |
| Medical health professions | 158 | 105 | 26 | 24.3 (16.2–34.5) | 1.1 (0.7–1.5) | 460 | 326 | 44 | 13.1 (9.8–17.2) | 0.6 (0.4–0.8) |
| Non–medical health professions, personal care, medical technology | – | | | | | 121 | 79 | 13 | 17.1 (10.0–27.4) | 0.8 (0.4–1.2) |
| Education, social and domestic professions, theology | 64 | 38 | 9 | 26.9 (13.9–44.9) | 1.1 (0.6–2.0) | 255 | 166 | 19 | 12.1 (7.8–18.2) | 0.5 (0.4–0.8) |
| Teaching professions | 195 | 114 | 30 | 28.9 (20.8–38.6) | 1.3 (0.9–1.7) | 264 | 189 | 30 | 21.7 (16.6–27.8) | 1.0 (0.7–1.2) |
| **Humanities, culture, design** | **203** | **136** | **32** | **24.6 (17.2–33.6)** | **1.1 (0.8**–**1.5)** | **191** | **142** | **15** | **10.7 (6.3–17.4)** | **0.5 (0.3–0.8)** |
| Advertising, marketing, commercial and editorial media professions | 120 | 82 | 16 | 20.8 (12.2–32.7) | 0.9 (0.5–1.5) | 129 | 94 | 13 | 13.8 (7.8–23.0) | 0.6 (0.4–1.0) |
| Product design, handicrafts | 26 | 16 | 6 | 35.6 (14.9–62.6) | 1.7 (0.7–2.8) | – | | | | |
| Performing and entertainment professions | 48 | 31 | 6 | 20.4 (7.8–41.9) | 0.9 (0.4–1.9) | – | | | | |
