# Supplementary material for: Incidence of type 2 diabetes and metabolic syndrome by Occupation – 10-Year follow-up of the Gutenberg Health Study
Source: BMC Public Health. 2025 Feb 7;25:502. doi: 10.1186/s12889-025-21732-5 (PMC11803924; doi:10.1186/s12889-025-21732-5)
Supplement: Supplementary file 4 — Supplementary Material 4. [file 12889_2025_21732_MOESM4_ESM.docx]

**Additional file 3:**

**Incidence of Type 2 Diabetes and Metabolic Syndrome by Occupation – 10-Year Follow-up of the Gutenberg Health Study**

Age- and sex-standardised incidence and SIR of type 2 diabetes for occupational areas and main occupational groups, stratified by sex

|  | **Men** | | | | | **Women** | | | | |
| --- | --- | --- | --- | --- | --- | --- | --- | --- | --- | --- |
| **Occupation** | **N** | **Persons**  **at risk** | **Incident cases** | **Age- and sex-standardised Incidence**  **(95% CI)** | **SIR (95% CI)** | **N** | **Persons**  **at risk** | **Incident cases** | **Age- and sex-standardised Incidence**  **(95% CI)** | **SIR (95% CI)** |
| **Agriculture, forestry, animal husbandry and horticulture** | **170** | **126** | **9** | **7.7 (3.8–14.4)** | **1.1 (0.6–2.1)** | **–** | | | | |
| Agriculture, animal husbandry and forestry | 111 | 86 | 8 | 9.7 (4.6–18.6) | 1.4 (0.7–2.7) | – | | | | |
| **Raw material extraction, production and manufacturing** | **931** | **672** | **54** | **8.1 (6.1–10.8)** | **1.2 (0.9–1.6)** | **164** | **103** | **8** | **12.2 (7.0–20.1)** | **1.7 (1.0–2.9)** |
| Metal production, processing and construction | 74 | 45 | 5 | 10.7 (3.6–25.9) | 1.5 (0.5–3.8) | – | | | | |
| Machine and vehicle technology | 258 | 184 | 21 | 10.9 (6.6–17.1) | 1.6 (1.0–2.5) | – | | | | |
| Mechatronics, energy and electronics | 186 | 135 | 7 | 4.3 (1.6–10.2) | 0.6 (0.2–1.5) | – | | | | |
| Technical development, design and production scheduling | 207 | 164 | 10 | 6.6 (3.3–12.5) | 0.9 (0.5–1.8) | – | | | | |
| Food production and processing | 55 | 37 | 6 | 20.8 (9.4–38.7) | 3.0 (1.4–5.6) | – | | | | |
| **Construction, architecture, surveying and building technology** | **366** | **264** | **22** | **10.2 (6.8–14.8)** | **1.5 (1.0–2.2)** | **–** | | | | |
| Construction planning, architecture, surveying | 109 | 83 | 5 | 7.6 (3.2–16.2) | 1.1 (0.5–2.4) | – | | | | |
| Building and supply services | 145 | 101 | 11 | 12.1 (6.6–20.7) | 1.8 (1.0–3.0) | – | | | | |
| **Natural Science, geography and computer science** | **445** | **348** | **24** | **6.3 (3.9–9.9)** | **0.9 (0.6–1.4)** | **104** | **87** | **6** | **10.7 (5.4–19.7)** | **1.5 (0.8–2.9)** |
| Mathematics, biology, chemistry, physics | 101 | 75 | 6 | 7.7 (2.9–17.8) | 1.1 (0.4–2.6) | 55 | 46 | 5 | 18.2 (9.0–32.6) | 2.8 (1.3–4.7) |
| Computer science, information and communications technologies | 328 | 263 | 18 | 6.5 (3.8–10.8) | 1.0 (0.6–1.6) | – | | | | |
| **Transport, logistics, protection and safety** | **533** | **338** | **35** | **10.4 (7.3–14.5)** | **1.5 (1.1–2.1)** | **183** | **110** | **9** | **7.3 (3.5–14.2)** | **1.0 (0.5–2.1)** |
| Traffic, logistics (except vehicle driver) | 205 | 121 | 9 | 8.3 (4.1–15.6) | 1.2 (0.6–2.3) | – | | | | |
| Drivers and mobile plant operators | 194 | 122 | 20 | 15.5 (9.5–24.1) | 2.3 (1.4–3.5) | – | | | | |
| Cleaners |  | – | | | | 63 | 31 | 5 | 13.6 (4.9–30.6) | 2.1 (0.7–4.4) |
| **Commercial services, trade in goods, sales, hotel and tourism** | **352** | **274** | **18** | **7.5 (4.7–11.7)** | **1.1 (0.7–1.7)** | **449** | **304** | **21** | **7.9 (5.4–11.4)** | **1.2 (0.8–1.7)** |
| Purchasing, sales and distribution, trade | 196 | 158 | 8 | 6.5 (3.3–12.1) | 0.9 (0.5–1.8) | – | | | | |
| Sales workers (retail) | 111 | 85 | 5 | 6.2 (2.3–14.6) | 0.9 (0.3–2.1) | 266 | 165 | 13 | 8.5 (5.2–13.7) | 1.2 (0.8–2.0) |
| Tourism, hotel and restaurant | 45 | 31 | 5 | 16.0 (5.9–34.7) | 2.4 (0.9–5.0) | 100 | 77 | 7 | 10.7 (5.4–19.6) | 1.5 (0.8–2.8) |
| **Business organisation, accounting, law and public administration** | **1007** | **760** | **63** | **9.1 (7.1–11.5)** | **1.3 (1.0–1.7)** | **1394** | **1039** | **56** | **5.7 (4.5–7.3)** | **0.8 (0.7–1.1)** |
| Corporate management and business organisation | 497 | 383 | 27 | 7.4 (5.0–10.9) | 1.1 (0.7–1.6) | 760 | 568 | 30 | 6.1 (4.4–8.4) | 0.9 (0.6–1.2) |
| Financial services, accounting, tax consultancy | 274 | 211 | 18 | 9.7 (6.0–15.1) | 1.4 (0.9–2.2) | 301 | 229 | 11 | 4.3 (2.2–7.8) | 0.6 (0.3–1.1) |
| Law and administration | 236 | 166 | 18 | 12.2 (7.8–18.6) | 1.7 (1.1–2.7) | 333 | 242 | 15 | 6.2 (3.7–10.2) | 0.9 (0.5–1.5) |
| **Health, social affairs, teaching and Education** | **458** | **330** | **21** | **7.1 (4.6–10.6)** | **1.0 (0.7–1.5)** | **1095** | **831** | **26** | **2.9 (2.0–4.3)** | **0.4 (0.3–0.6)** |
| Medical health professions | – | | | | | 458 | 349 | 8 | 2.6 (1.3–4.9) | 0.4 (0.2–0.7) |
| Education, social and domestic professions, theology | – | | | | | 254 | 187 | 10 | 4.6 (2.3–8.9) | 0.7 (0.3–1.3) |
| Teaching professions | 195 | 136 | 10 | 8.2 (4.3–14.5) | 1.2 (0.6­–2.1) | 262 | 202 | 6 | 2.3 (0.9–5.4) | 0.4 (0.1–0.8) |
| **Humanities, culture, design** | **202** | **153** | **7** | **5.0 (2.1–10.7)** | **0.8 (0.3**–**1.6)** | **190** | **149** | **7** | **5.2 (2.4–10.5)** | **0.8 (0.4–1.5)** |
| Advertising, marketing, commercial and editorial media professions | – | | | | | 129 | 100 | 6 | 6.3 (2.6–13.6) | 0.9 (0.4–2.0) |
